# Accurate, Scalable Structural Variant Genotyping in Complex Genomes at Population Scales

**DOI:** 10.1093/molbev/msaf180

**Published:** 2025-07-29

**Authors:** Ming Hu, Penglong Wan, Chengjie Chen, Shuyuan Tang, Jiahao Chen, Liang Wang, Mahul Chakraborty, Yongfeng Zhou, Jinfeng Chen, Brandon S Gaut, J J Emerson, Yi Liao

**Affiliations:** Key Laboratory of Biology and Genetic Improvement of Horticultural Crops (South China), Ministry of Agriculture and Rural Affairs, College of Horticulture, South China Agricultural University, Guangdong 510642, China; Key Laboratory of Biology and Genetic Improvement of Horticultural Crops (South China), Ministry of Agriculture and Rural Affairs, College of Horticulture, South China Agricultural University, Guangdong 510642, China; Tropical Crops Genetic Resources Institute, Chinese Academy of Tropical Agricultural Sciences, State Key Laboratory of Tropical Crop Breeding, Laboratory of Crop Gene Resources and Germplasm Enhancement in South China, Ministry of Agriculture and Rural Affairs, Key Laboratory of Tropical Crops Germplasm Resources Genetic Improvement and Innovation of Hainan Province, Hainan 571101, China; Key Laboratory of Biology and Genetic Improvement of Horticultural Crops (South China), Ministry of Agriculture and Rural Affairs, College of Horticulture, South China Agricultural University, Guangdong 510642, China; Key Laboratory of Biology and Genetic Improvement of Horticultural Crops (South China), Ministry of Agriculture and Rural Affairs, College of Horticulture, South China Agricultural University, Guangdong 510642, China; Key Laboratory of Biology and Genetic Improvement of Horticultural Crops (South China), Ministry of Agriculture and Rural Affairs, College of Horticulture, South China Agricultural University, Guangdong 510642, China; Department of Biology, Texas A&M University, College Station, TX 77843, USA; Tropical Crops Genetic Resources Institute, Chinese Academy of Tropical Agricultural Sciences, State Key Laboratory of Tropical Crop Breeding, Laboratory of Crop Gene Resources and Germplasm Enhancement in South China, Ministry of Agriculture and Rural Affairs, Key Laboratory of Tropical Crops Germplasm Resources Genetic Improvement and Innovation of Hainan Province, Hainan 571101, China; State Key Laboratory of Integrated Management of Pest Insects and Rodents, Institute of Zoology, Chinese Academy of Sciences, Beijing 100101, China; Department of Ecology and Evolutionary Biology, University of California, Irvine, CA 92697, USA; Department of Ecology and Evolutionary Biology, University of California, Irvine, CA 92697, USA; Key Laboratory of Biology and Genetic Improvement of Horticultural Crops (South China), Ministry of Agriculture and Rural Affairs, College of Horticulture, South China Agricultural University, Guangdong 510642, China

**Keywords:** structural variation, population genotyping, pangenome, comparing methods, plant genome

## Abstract

Comparisons of complete genome assemblies offer a direct procedure for characterizing all genetic differences among them. However, existing tools are often limited to specific aligners or optimized for specific organisms, narrowing their applicability, particularly for large and repetitive plant genomes. Here, we introduce Structural Variants Genotyping of Assemblies on Population scales (SVGAP), a pipeline for structural variant (SV) discovery, genotyping, and annotation from high-quality genome assemblies at the population level. Through extensive benchmarks using simulated SV datasets at individual, population, and phylogenetic contexts, we demonstrate that SVGAP performs favorably relative to existing tools in SV discovery. Additionally, SVGAP is one of the few tools to address the challenge of genotyping SVs within large assembled genome samples, and it generates fully genotyped VCF files. Applying SVGAP to 26 maize genomes revealed hidden genomic diversity in centromeres, driven by abundant insertions of centromere-specific LTR-retrotransposons. The output of SVGAP is well-suited for pangenome construction and facilitates the interpretation of previously unexplored genomic regions.

## Background

Structural variants (SVs) are commonly defined as genome alterations between individuals in either the order or content of DNA spanning 50 or more base pairs (bp) (Alkan et al. 2011; Gaut et al. 2018; Mahmoud et al. 2019). These include simple variants like deletions, insertions, duplications, translocations, or inversions, as well as more complex DNA rearrangements (Escaramís et al. 2015; Hadi et al. 2020; Li et al. 2020b ). Despite being recognized as major sources of functional variation for over a century (Sturtevant 1913; Bridges 1936), SVs are still the least well-characterized genetic variants, especially in plants (Saxena et al. 2014; Kou et al. 2020; Yuan et al. 2021). Only recently has a growing emphasis been placed on genome-wide analyses of SVs, driven by significant advancements in detection technologies (Liu et al. 2020; Hufford et al. 2021; Qin et al. 2021; Zhou et al. 2022; Chen et al. 2023b, 2023d). These studies highlight the important roles that SVs play in processes ranging from mutation to evolution (Carvalho and Lupski 2016; Schneider et al. 2016; Stuart et al. 2023), from ecology to speciation, and from human health and survival to crop genome diversity, domestication, migration history, breeding, and traits (Fuentes et al. 2019; Gao et al. 2019; Liu et al. 2020; Li et al. 2022). Furthermore, SVs impact more of the genome than single-nucleotide variations (SNVs) (Hämälä et al. 2021), and recent studies demonstrated that incorporating SVs into pangenome graphs significantly enhances the power of genome-wide association studies, captures missing heritability, and empowers crop breeding (Zhou et al. 2022). Comprehensive and accurate SV discovery and genotyping are required for the effective study of these important issues.

Detection and analysis of SVs have greatly benefited from the rapid advancement of sequencing technologies. While early studies (e.g. those employing cytogenetic approaches or microarray assays) allowed us to probe variation in genome structure, sequencing-based methods have the potential to identify both large and small SVs at a bp resolution, can probe any region that can be sequenced, and, perhaps most importantly, permit studies to scale to large sample sizes quickly and inexpensively. Sequencing methods can be broadly categorized into three major groups: short-read reference mapping, long-read reference mapping, and whole-genome assembly-based methods (Mahmoud et al. 2019). To date, nearly 80 pieces of software based on short-read sequencing and 50 based on long-read sequencing have been developed (Kosugi et al. 2019; Ahsan et al. 2023). They adopt diverse algorithms for resolving different types of sequencing platforms and experimental scenarios. Benchmark analyses reveal that no single tool achieves optimal performance for SV detection, with trade-offs between accuracy and completeness being common (Cameron et al. 2019; Liu et al. 2024). Furthermore, benchmark experiments based on simulated data in animals revealed that short-read-based methods capture only a small proportion of SVs (i.e. ∼50%) (Cleal and Baird 2022), while long-read-based methods achieve a higher but still incomplete rate of between 70% and 84% (Dierckxsens et al. 2021). Benchmark analyses in plants are relatively rare compared to animals. However, due to their highly repetitive and complex nature, it seems unlikely that performance in plant genomes will exceed performance in other organisms. Indeed, SV detection continues to pose significant challenges. Researchers are actively developing new tools employing cutting-edge strategies and algorithms, such as pangenomics (Hickey et al. 2020; Chin et al. 2023) and machine learning (Lin et al. 2022; Popic et al. 2023), aiming to achieve a comprehensive description of the entire landscape of SVs across all genomic contexts (Denti et al. 2023).

A de novo assembly-based strategy is widely anticipated to be a superior approach for characterizing SVs (Chen et al. 2023e). Its ability to identify all differences between two haplotypes is theoretically limited only by the ability to accurately assemble the haplotypes (Ebert et al. 2021). This capability has recently seen dramatic improvements in a very short time. Such dramatic advances are already providing access to previously unexplored genomic contexts, including challenging centromeric regions (Logsdon et al. 2024). Numerous studies have uncovered a significant proportion of hidden SVs that are missed by short-read and even long-read-based methods (Chakraborty et al. 2018). However, the extensive effort and cost required to generate high-quality genome assemblies have often led to the neglect of this approach. Fortunately, sequencing technologies and computational methods have become increasingly feasible and cost-effective, making reference-grade genome assemblies far more accessible (De Coster et al. 2021). Consequently, population-scale high-quality genome assemblies are now widely attainable and are becoming routine parts of the toolkit of biologists studying genetic variation (Hufford et al. 2021). In this context, assembly-based variant calling and joint genotyping methods have emerged as attractive options for SV detection, offering indispensable benefits in genetic discovery and serving as valuable complements to pangenome construction (Cochetel et al. 2023; Wang et al. 2023).

There are several methods available for calling SVs by aligning assembled genomes to a reference (Ahsan et al. 2023). These methods typically require a prealignment process before SV calling. Some methods, such as SyRI (Goel et al. 2019), MUM&Co (O’Donnell and Fischer 2020), SVMU (Chakraborty et al. 2018), and Assemblytics (Nattestad and Schatz 2016), rely on whole-genome alignments (WGAs) from specific aligners. Others, such as Dipcall (Li et al. 2018), SVIM-asm (Heller and Vingron 2021), presence and absence variants (PAV) (Ebert et al. 2021), and cuteSV (Jiang et al. 2020), may also employ large contig alignments. Additionally, certain aligners themselves, like minimap2 (Li 2018), GSAlign (Lin and Hsu 2020), and AnchorWave (Song et al. 2022), offer the option to call SVs during the alignment process. However, it is worth noting that most of these SV callers were developed and tested on a narrow range of taxa (typically animal genomes), which naturally raises questions about their effectiveness in plants. Plant genome structures are more diverse than mammalian genomes, often exhibiting variation in ploidy, greater sequence diversity, extensive rearrangements, and a high density of repetitive sequences (Zhao and Schranz 2019; Song et al. 2024 ).

When developing a de novo assembly-based SV discovery method, three key aspects should be taken into account. First, since this method relies on WGAs as input, it is essential to validate the performance of alignment tools on the genomes being analyzed. Second, a comprehensive set of SV “truth sets” should be employed for benchmarking purposes. Finally, the tools developed should be suitable for application across population samples and leverage population-level data to enhance genotyping; to our knowledge, no such tools currently exist.

In this work, we introduce Structural Variants Genotyping of Assemblies on Population scales (SVGAP), a flexible pipeline to detect, genotype, and annotate SVs in large samples of de novo genome assemblies. It compares each sample to a reference genome in WGAs to call SVs. The SVs identified are subsequently combined across samples to generate a nonredundant call set. Each SV call in this call set can be further regenotyped by examining local alignment information specific to each sample to produce fully genotyped Variant Call Format (VCF) files. The pipeline can optionally be applied to detect and report small variants such as indels and single nucleotide polymorphisms (SNPs). SVGAP categorizes SVs according to their mutation class, including tandem duplications, transposable element (TE) insertions, or gene translocations, among others. This annotation adds valuable information to the detected SVs, enhancing the understanding of their potential biological impact.

To ensure the wide applicability and feasibility of SVGAP, we conducted thorough testing on a diverse range of commonly used aligners for WGAs. We specifically included the aligners that exhibited excellent performance in the SVGAP pipeline, ensuring compatibility with genomes of varying complexity, particularly in plants. Through comprehensive benchmarking analyses using simulated SVs at individual, population, and phylogenetic levels, we show that SVGAP surpasses existing tools in terms of accuracy and completeness, regardless of SV type, sequence divergence, and genomic regions. Our benchmarking quantifies the accuracy of SV genotyping conducted by SVGAP. To evaluate SVGAP's feasibility for real data, we applied it to 26 maize genomes, highlighting its proficiency in detecting SVs at all genomic texts. The resulting VCF files can be used for pangenome construction and facilitate the genomic analysis of previously inaccessible genomic contexts. SVGAP is implemented in Perl and is freely available under the MIT license at https://github.com/yiliao1022/SVGAP.

## Results

### Overview of SVGAP

#### Conceptual Framework and Detailed Functionality

Detecting SVs from WGAs presents two primary challenges: distinguishing orthologous from paralogous alignments and identifying large SVs from fragmented alignment blocks. SVGAP seeks to address these issues by constructing alignment chains and nets using University of California Santa Cruz (UCSC) tools (Kent et al. 2003). Chains represent collections of colinear local alignments between two genomes, while nets hierarchically organize the highest-scoring nonoverlapping chains to form a genome-wide, single-coverage alignment framework. Gaps within chains or nets often reflect underlying SVs. This framework enables the accurate detection of large SVs, including those in syntenic regions and complex rearranged loci such as inversions and translocations (supplementary fig. S1, Supplementary Material online).

SVGAP identifies various types of SVs by analyzing alignment nets. These include small insertions and deletions (<50 bp), large insertions and deletions (≥50 bp), tandem duplications, inversions, translocations, and complex genomic loci where the reference and query genomes fail to align, resulting in double-sided gaps. To enhance specificity, SVGAP offers an option to filter out potential paralogous alignments, retaining only the top-scoring or most confident chains for SV calling. This filtering is customizable and can be adapted to the genomic complexity of different datasets.

As SVs are detected individually for each sample, SVGAP provides a merging function to generate a nonredundant SV call set. To identify putatively identical events, SVGAP first combines calls of the same SV type across samples and sorts them based on coordinates. Subsequently, different strategies are employed for each type of SV. For example, deletions and inversions are merged using an adjustable threshold for overlap (e.g. 90%). If the coordinates of two SVs overlap by at least that threshold, they are considered the same event and merged. In the case of insertion events, sequence identity is also taken into account in addition to coordinates. This ensures not only that the coordinates but also that the actual sequence of the inserted fragment is considered for identification and merging purposes.

After generating the nonredundant SV set, SVGAP proceeds to regenotype each call across all samples using the corresponding filtered pairwise WGA. This involves genotyping each SV site in every sample by extracting and examining the local sequence alignments. SVGAP also offers a program to genotype SNVs using the filtered one-to-one alignment files. The outcome of this step is fully genotyped VCF files for each SV type, as well as for SNVs. These files are well-suited for further pangenome construction and evolutionary population genetics studies.

SVGAP also aims to annotate SVs by inferring the mechanisms underlying their formation. In other words, SVGAP not only identifies alignment gaps as indels but also interprets their biological origins. First, it can recognize an insertion or deletion as a duplication or contraction derived from flanking sequences by comparing the inserted or deleted sequences to their immediate context. Second, SVGAP can detect TE insertions by comparing SV sequences to a TE library. Third, it can identify whether an insertion represents a gene duplication. This biologically informed approach improves the accurate characterization of complex insertions, including those involving multiple TE fragments.

#### Workflow and Pipeline Overview

**Fig. 1.**
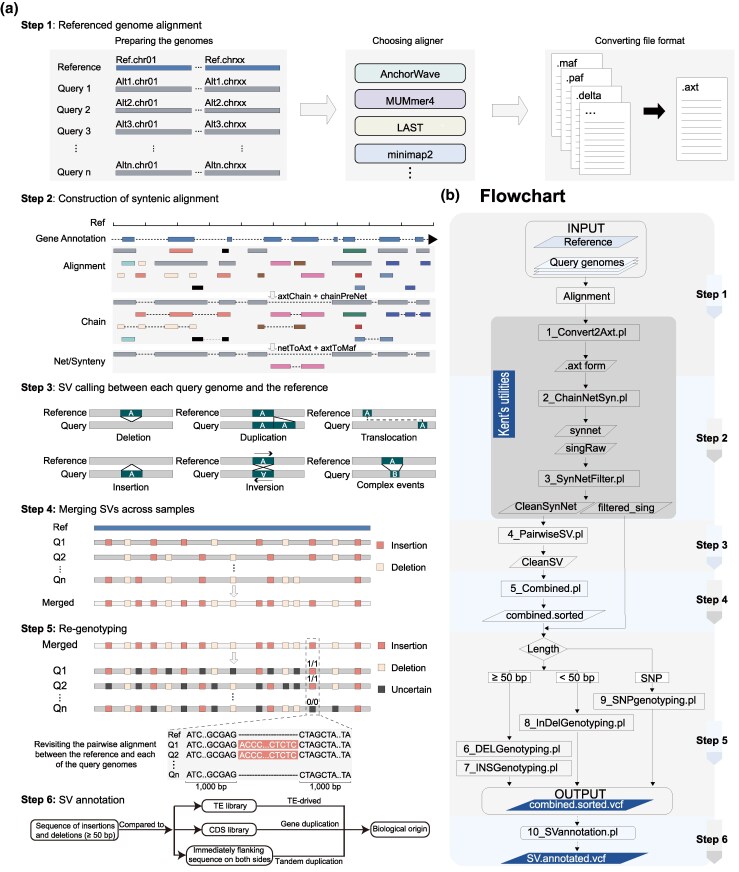
Overview of the SVGAP workflow. a) The six main steps in the SVGAP pipeline: step 1: converting WGA results from external alignment tools into the desired AXT format files; step 2: employing Kent's utilities to construct syntenic alignments at chromosome-scale level; step 3: identifying SVs between each sample and the reference; step 4: merging SVs across samples to generate a unique SV call set; step 5: regenotyping SVs and producing the fully genotyped VCF files; and step 6: annotating SVs for understanding mechanisms underlying their formation. b) The flowchart and Perl scripts implemented in each step of the SVGAP pipeline.

The SVGAP workflow consists of six main steps (Fig. 1a): (1) alignment preprocessing, (2) synteny chain and net construction, (3) SV detection, (4) SV merging across samples, (5) regenotyping of SVs, and (6) functional annotation. These steps are executed sequentially using a set of Perl programs (Fig. 1b). In steps 1–3, each sample is independently compared to the reference genome, allowing for parallelized processing. SVGAP supports a variety of genome alignment tools (e.g. LAST, MUMmer, minimap2, and AnchorWave), and their outputs are converted into the standardized AXT format used by UCSC tools. This ensures broad compatibility and allows users to apply their preferred aligner while fully utilizing SVGAP's downstream functionality. A detailed usage guide is available in Supplementary note S1, Supplementary Material online. In the following sections, we present benchmarking results across multiple datasets and compare SVGAP to existing tools, highlighting its accuracy, efficiency, and utility for population-scale structural variation analysis.

### Assessing Aligners for WGA of Plant Genomes

Although WGAs serve as input for assembly-based SV identification approaches, the consequences of alignment software choice have yet to see much attention, especially in the case of plants. To fill this gap, we conducted a preliminary assessment of 14 commonly used aligners by applying them to two rice (*Oryza sativa*) genomes (∼380 Mb), MH63 and ZS97 (Song et al. 2021). We evaluated each aligner for various metrics, including computational speed, memory usage, and alignment quality. Based on these results, we retained six aligners for further consideration: Lastz, Last, MUMmer4, AnchorWave, GSAlign, and minimap2. Details for running these tools are described in supplementary note S2, Supplementary Material online.

**Fig. 2.**
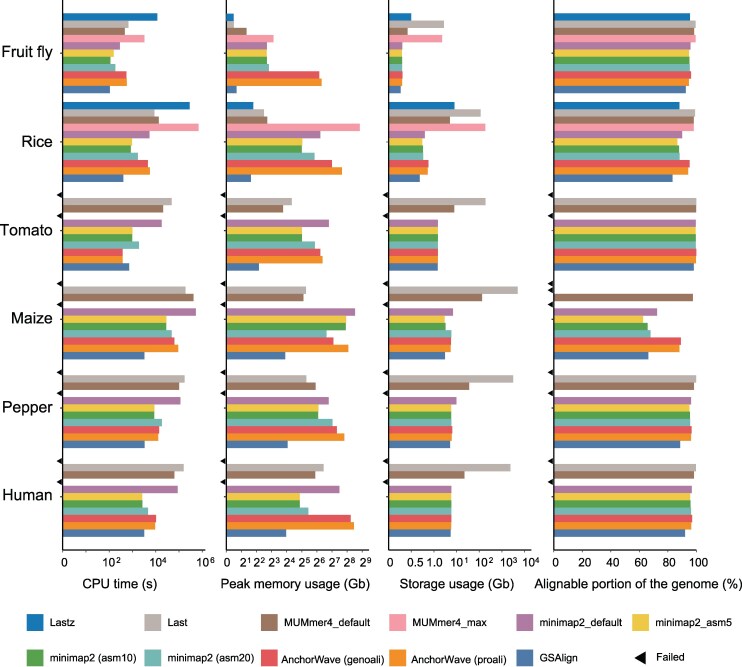
The performance of six widely used WGA tools on genomes of *Drosophila*, rice, tomato, maize, pepper, and human, representing varying levels of complexity. Metrics assessed include a) runtime, b) peak memory consumption, c) volume of raw alignments generated, and d) percent coverage of the reference. Each aligner's performance was measured by aligning two representative genomes within each species as shown in supplementary table S3, Supplementary Material online. Black triangles indicate cases where a given aligner failed to complete alignment for a particular species.

We next expanded the assessment of the six selected aligners to larger and more repetitive plant genomes (i.e. tomato, maize, and pepper), representing a diverse range of genome complexities (supplementary table S1, Supplementary Material online). We also included fruit fly (*Drosophila melanogaster*) and human (*Homo sapiens*) genomes for comparison. For each combination of species and aligner, we calculated pairwise alignments across a total of 11 aligner–parameter conditions (supplementary table S2, Supplementary Material online). Figure 2 illustrates the running time, memory usage, storage space requirements, and alignable portion of the genome for each aligner across the tested species (see the “Methods” section for more details on evaluation metrics and supplementary table S3, Supplementary Material online, for tool execution details). To summarize their performance, GSAlign, AnchorWave, and MUMmer4 completed the alignments for all species using their default parameters. However, minimap2 exceeded system memory capacity (NGB 1 TB) when aligning the maize and pepper genomes. Additionally, Lastz failed for these same two species as well as the tomato and human genomes due to excessive runtime. To address the memory capacity issue encountered when running minimap2 when aligning the maize and pepper genomes, we employed a solution by dividing the query genome into smaller segments (e.g. using a 20-Mb window with a 2-Mb step) before performing the alignment against the reference genome. Additionally, when using its default parameters, Last generated a significant amount of raw alignments for the human, pepper, and maize genomes (2.0, 3.1, and 5.1 Tb, respectively), which poses a challenge for downstream analysis. MUMmer4 only completed alignments for fly and rice genomes in the “-maxmatch” mode. Otherwise, all other alignments completed without issue and could be assessed. Overall, these results quantify the aligners' capacity to align genomes, particularly in the context of plants with varying levels of genome complexity. They also highlight the key practical challenges in aligning plant genomes. This information serves as a valuable guide for selecting aligners when developing de novo assembly-based methods for SV discovery.

### Performance of SVGAP Across Different Aligners

**Fig. 3.**
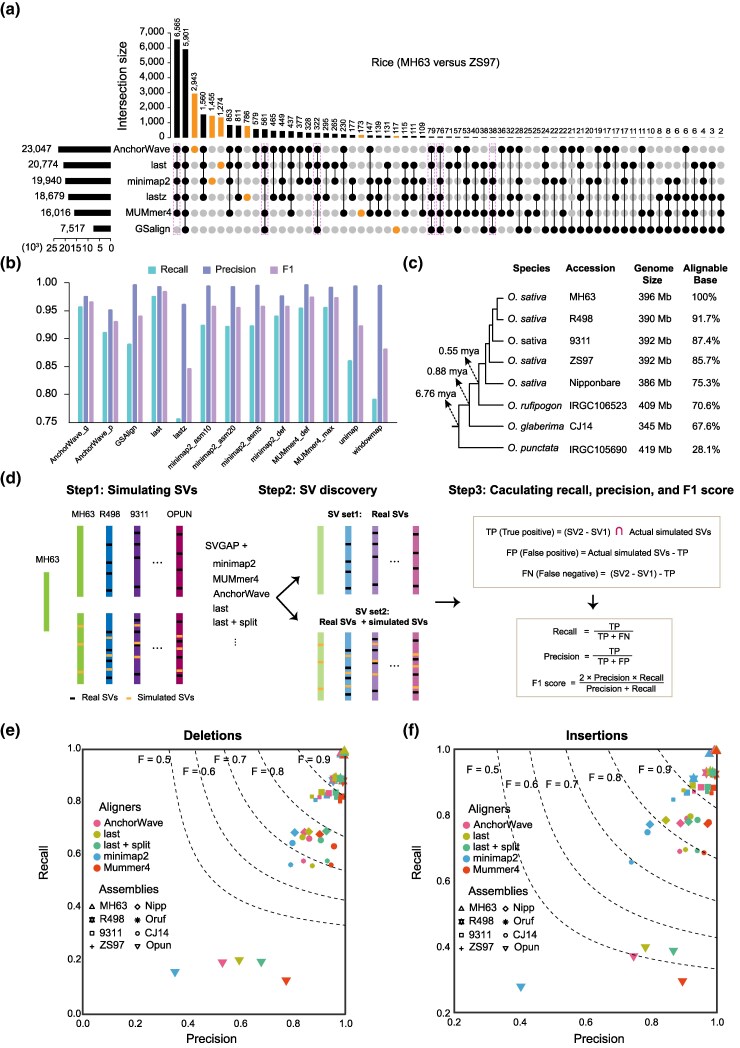
Performance of SVGAP for SV detection across different aligners. a) Analysis of shared SV calls—including deletions and insertions—among various aligners applied to two rice reference genomes. b) Comparative assessment of SVGAP performance in detecting SVs between a rice reference genome and its simulated counterpart, which includes introduced SVs. c) Phylogenetic relationships and estimated divergence times of selected rice (*Oryza*) genome assemblies, along with their approximate percentage of sequence alignment to the reference genome (MH63). d) Strategies for evaluating SVGAP performance across different aligners at varying levels of sequence divergence, along with the formulas used to calculate recall, precision, and F1 score. e and f) Comparison of SVGAP performance across aligners in detecting SVs, with panel e representing deletions and panel f insertions, at different levels of sequence divergence within the *Oryza* system.

We supplied the pairwise WGAs obtained from the six best aligners (i.e. AnchorWave, minimap2, Last, Lastz, MUMmer4, and GSAlign) to evaluate the performance of SVGAP by comparing the consistency and variability of SV calls across these aligners. Our analysis focused on deletions and insertions, which are the most common types of SVs, using genomes from the fly, rice, and tomato. For the rice genomes MH63 and ZS97 (Song et al. 2021), SVGAP detected a wide range of SVs (≥50 bp) depending on the aligner, with deletion counts ranging from 3,735 to 11,642 and insertion counts from 3,821 to 11,542. Among the aligners, AnchorWave reported the highest number of SV calls, followed by Last, minimap2, Lastz, MUMmer4, and GSAlign (supplementary table S4, Supplementary Material online). The consistency of SV calls from pairwise comparisons varied between 27.4% and 92.5% (supplementary fig. S2a, Supplementary Material online, for combined insertions and deletions). We used an upset plot (Fig. 3a, calculation details are provided in the “Methods” section) to visualize the consistency between different aligners. In total, these six aligners identified 28,548 unique insertions and deletions between the genomes MH63 and ZS97. Approximately 47.4% (13,542) of them were reported by at least five aligners, while nearly 23.6% (6,748) were reported by only one aligner. Among the aligner-specific SV calls, the proportion of AnchorWave calls was the highest, accounting for 12.78% (2,943/23,047) of its total calls. minimap2 reported 7.3% (1,455/19,940), Last reported 6.1% (1,274/20,774), Lastz reported 4.2% (786/18,679), GSAlign reported 1.6% (117/7,517), and MUMmer4 reported 1.1% (173/16,016). The majority of the aligner-specific SV calls—e.g. up to 94% for AnchorWave and 75% for minimap2—are well supported by gaps in their own sequence alignments (supplementary fig. S3, Supplementary Material online), likely reflecting the distinct alignment isoforms generated by each aligner. Notably, differences among aligners become more pronounced as genome complexity increases, as demonstrated by comparable analyses of the fly and tomato genomes (supplementary table S4 and fig. S2b–e, Supplementary Material online). These observations indicate that the choice of aligners significantly impacts SV detection.

To assess the performance of SVGAP on a validated truth set, we randomly simulated 7,264, 20,328, and 28,440 deletions and 4,290, 10,046, and 15,245 insertions (≥50 bp) using RSVSim (Bartenhagen and Dugas 2013) in the reference genomes of fly (iso-1), rice (Nipponbare), and tomato (SL4), respectively (supplementary table S5, Supplementary Material online). We simulated proportionally more deletions than insertions in each genome because mutational processes tend to favor deletions over insertions (Kuo and Ochman 2009). The reference genomes were then aligned with their respective simulated versions employing different aligners and parameter settings for SV detection. The resulting SV callsets were cross-referenced with the validated truth set to assess the precision of SV discovery by SVGAP in combination with the different aligners. These comparisons highlight that SVGAP combined with Last consistently outperformed all the other aligners, achieving the highest recall (97.5% to 99.0%), precision (99.0% to 99.7%), and F1 score (98.4% to 99.4%) for both insertions and deletions (≥50 bp) across all three species (supplementary table S5, Supplementary Material online, and example shown for rice in Fig. 3b). While other aligners also exhibited comparably high performance with SVGAP, their F1 scores ranged from 97.2% to 99.2% for MUMmer4, 95.1% to 98.1% for minimap2, 91.7% to 97.3% for AnchorWave, 93% to 97.1% for GSAlign, and 79.7% to 95% for Lastz. Notably, Lastz showed the poorest performance in plant genomes, suggesting it may not be an ideal choice for SV discovery within the SVGAP workflow when analyzing large and repetitive plant genomes.

We further evaluated the performance of SVGAP across different aligners at various levels of sequence divergence, excluding Lastz and GSAlign due to their inferior performance as previously demonstrated. To achieve this, we simulated SVs in genomes with varying phylogenetic distances from a reference genome. We then applied SVGAP with different aligners to identify SVs between each simulated genome and the reference, as well as between each original genome and the reference. The latter served as the background set of SVs, which were subsequently subtracted from the former. The resulting SV sets were used to calculate recall and precision, as illustrated in Fig. 3c. For instance, using MH63 (*O. sativa*) as the reference genome, we introduced 10,000 deletions (between 50 and 20,000 bp) and 10,000 insertions (between 50 and 40,000 bp) into MH63 itself and seven additional rice genome assemblies (see the “Methods” section). These additional assemblies included four from *O. sativa*, along with one assembly from each closely related species: *Oryza rufipogon*, *Oryza glaberrima*, and *Oryza punctata*. Collectively, these represented an estimated divergence span of 6.78 million years (Stein et al. 2018), with the percentage of syntenic sequence alignment to the reference ranging from 28.1% to 100% (Fig. 3d; see the “Methods” section for calculation). Following the aforementioned strategy (Fig. 3d), we demonstrated that SVGAP successfully detected SVs across diverged genomes with all four tested aligners (Last, MUMmer4, minimap2, and AnchorWave), yielding similar levels of recall and precision (Fig. 3e, 3f, and supplementary table S6 and fig. S4, Supplementary Material online). Among these, Last and MUMmer4 consistently outperformed the other aligners, likely due to their specialized design for WGAs. Similar results were also observed in tomatoes (supplementary fig. S5, Supplementary Material online). These findings highlight the ability of SVGAP to accurately identify SVs between diverse and repetitive plant genomes using various aligners.

### Comparison of SVGAP With Other Genome Assembly-Based SV Callers

**Fig. 4.**
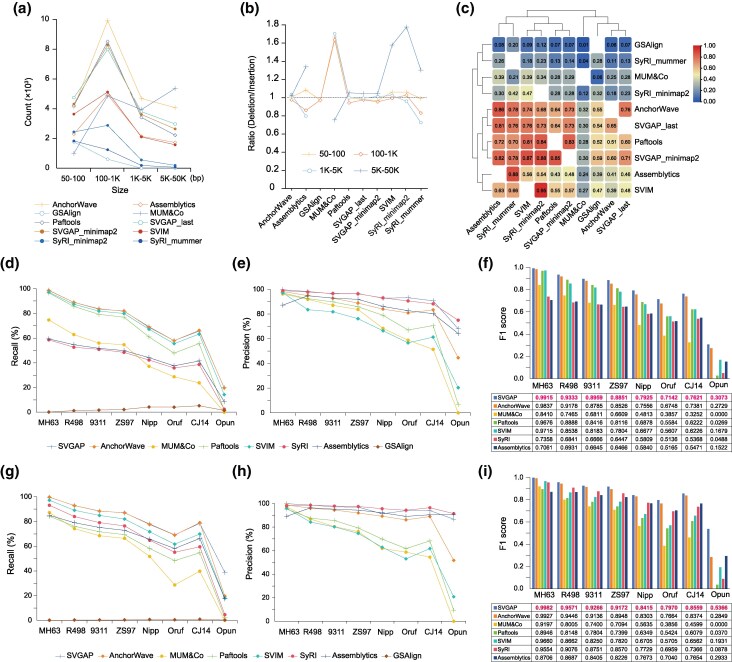
Performance comparison of SVGAP with other methods. a) Number of SV calls reported by different methods across various length ranges when comparing the two rice genomes, MH63 and ZS97. b) Ratio of deletions to insertions reported by different methods, again based on the two rice genomes. c) Overlap of SVs in pairwise comparisons among different methods. d) Recall for the benchmark analysis of deletions when comparing MH63 with eight other divergent genomes, including simulated SVs. e) Precision of deletion detection. f) F1 score for deletions. g) Recall for the analysis of insertions. h) Precision of insertion detection. i) F1 score for insertions.

We compared SVGAP (based on last alignments) to seven widely SV callers—Assemblytics (Nattestad and Schatz 2016), SyRI (Goel et al. 2019), SVIM-asm (Heller and Vingron 2021), paftools (Li 2018), GSAlign (Lin and Hsu 2020), MUM&Co (O’Donnell and Fischer 2020), and AnchorWave (Song et al. 2022)—all of which support the assembly-vs.-assembly strategy for SV detection. To evaluate their performance on real data, we applied each tool to identify SVs between two rice genomes, MH63 and ZS97 (see supplementary table S7, Supplementary Material online, for parameters). For simplicity and consistency, we focused only on deletions and insertions, as these are the primary SV types detected by all tools. Among the evaluated methods, AnchorWave identified the highest number of SVs (24,960), followed by SVGAP (21,241), paftools (19,889), MUM&Co (19,514), SVIM-asm (13,402), Assemblytics (11,993), and SyRI (6,571), while GSAlign detected the fewest SVs (2,557) (supplementary table S8, Supplementary Material online). In addition to large differences in the total number of calls, some methods exhibited distinct biases in SV size and type. For example, MUM&Co identified significantly more large SVs (>5 kb), whereas SyRI and GSAlign reported few, if any, SVs exceeding 1 kb (Fig. 4a). Furthermore, with the exception of AnchorWave, SVGAP, and paftools, all other tools showed a bias toward either deletions or insertions (Fig. 4b). These results reveal substantial variability in detection efficiency and specificity across tools on real data.

We also evaluated the concordance and discrepancies of SV calls across different methods through calculating the overlap rate in pairwise comparisons. This overlap rate was defined as the proportion of SVs from one method that had a reciprocal overlap of at least 80% with the SV calls from another method. Our analysis revealed a wide range of concordance rates between pairs of methods, reflecting both shared and unique SV detections (Fig. 4c). Overall, AnchorWave, SVGAP, and paftools had higher overlaps with other methods and with each other, followed by SVIM and Assemblytics. In contrast, MUM&Co, GSAlign, and SyRI showed much lower overlaps with other tools, mainly due to their smaller number of SV calls. Similar results were also obtained in comparing two tomato genomes: SL4 and M82 (Alonge et al. 2020) (supplementary fig. S6a–c, Supplementary Material online). These results highlight the challenge of reconciling SV datasets generated by diverse tools.

We next benchmarked SVGAP against other tools using simulated SVs across genomes (*n* = 8) with varying levels of sequence divergence in rice and tomato. These simulated genomes were described in the previous section (in Fig. 3c for rice and in supplementary fig. S5a, Supplementary Material online, for tomato). This allowed us to systematically evaluate the effectiveness of each method in detecting SVs as divergence increased. Since GSAlign reported very few SVs during testing, it was excluded from further comparisons. When identifying SVs between the reference genome and its modified version—representing zero sequence divergence—SVGAP consistently achieved the highest F1 scores across SV types and species, with at least 99.15% for rice and 99.53% for tomato (supplementary table S6, Supplementary Material online). As sequence divergence increased, SVGAP continued to yield the highest F1 scores for both deletions (Fig. 4d–f) and insertions (Fig. 4g–i) across all divergent rice genomes, while also maintaining superior recall and precision. AnchorWave showed the second-best performance. A similar trend was observed in the tomato datasets (supplementary fig. S6d–g, Supplementary Material online). These results indicate that SVGAP outperforms existing methods in SV discovery across a wide range of sequence divergence.

### SVGAP Enables SV Genotyping in Large Samples of Assembled Genomes

SVGAP independently identifies SVs for each individual sample. The SV calls from multiple samples are then merged to create a unique, nonredundant set of SVs. However, this merged set initially lacks complete genotype information, as it only includes positive calls (indicating the presence of variants). To address this limitation, SVGAP provides tools for regenotyping all SV calls across samples. This regenotyping process examines pairwise alignments of each genome against the reference, extracting local sequence alignments around the SV breakpoints—specifically targeting regions extending a user-defined length (e.g. 1 kb) upstream and downstream. By revisiting these local alignments, the tool infers the genotype of each SV in every sample, classifying them as positive, negative, or missing. A more detailed schematic of this process is shown in supplementary fig. S7, Supplementary Material online.

**Fig. 5.**
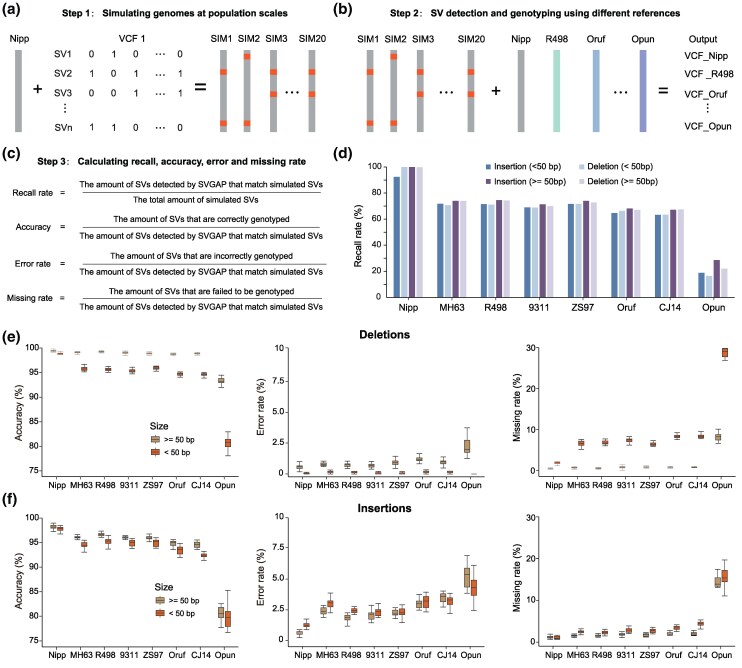
Benchmark analysis of merging and genotyping functions in SVGAP. a) Strategies for simulating population-scale genomes. b) SV discovery among population-scale genomes when using eight divergent genomes as the references. c) Formulas for computing recall rate, accuracy, error rate, and missing rate. d) Recall comparing the reference genome Nipponbare with eight other rice genomes across varying divergence scales, categorized by length. e) Accuracy, error rate, and missing rate for deletions. f) Accuracy, error rate, and missing rate for insertions across sequence divergence.

To evaluate the performance of SVGAP's merging and genotyping steps, we conducted benchmarking using population-scale simulated genome assemblies from two plant species: rice and tomato. For rice, simulated genomes were generated by randomly introducing SVs into four chromosomes of the reference genome “Nipponbare.” These SVs were coordinate-shifted versions of a ground-truth dataset comprising simple SVs—including insertions, deletions, and TE-induced insertions—originally identified from comparisons between “Nipponbare” and 48 diverse rice accessions (see the “Methods” section). We generated 20 simulated samples, each containing approximately half of the SVs, along with one sample containing the full set (Fig. 5a). After applying SVGAP to identify SVs relative to the reference genome, the merging step successfully recovered 99.8% (3,074 out of 3,079) of all simulated SVs (≥50 bp), producing only three false positives (FPs) (Fig. 5b; supplementary table S9, Supplementary Material online). Genotyping also showed high accuracy, with an average of 99.0% of deletions and 98.2% of insertions correctly genotyped across the 20 samples. The average error rate was 0.59% for insertions and 0.61% for deletions, while the missing rate was 1.21% for insertions and 0.39% for deletions (supplementary table S10, Supplementary Material online). These results demonstrate that SVGAP can accurately merge and genotype nearly all SVs with minimal errors in genomes exhibiting low levels of sequence divergence. Comparable performance was observed in the tomato datasets (supplementary tables S9 and S10, Supplementary Material online).

To further assess the impact of sequence divergence on the merging and genotyping processes, we repeated the analysis by identifying SVs in the 20 simulated rice genome assemblies using 7 additional, increasingly divergent rice genomes as references (see Fig. 3c). As expected, retrieval efficiency—or recall (as defined in Fig. 5c)—declined with increasing sequence divergence (Fig. 5d), primarily due to a reduction in alignable regions between genomes. After applying a rough normalization based on the alignable proportion—i.e. dividing recall by the estimated alignable fraction—SVGAP still achieved nearly 90% recall, indicating that it successfully detected the majority of simulated SVs located within alignable regions across divergent genomes (supplementary table S9, Supplementary Material online). For genotyping, we evaluated only those SVs that were successfully retrieved and found that accuracy also decreased with increasing divergence, particularly for smaller events (<50 bp) and insertions (Fig. 5e and 5f). With the exception of *O. punctata*, whose genome shares only ∼28% syntenic alignment with “Nipponbare,” SVGAP achieved genotyping accuracies of at least 97.9% for deletions and 94.5% for insertions (≥50 bp) across all other references (Fig. 5e and 5f; supplementary table S10, Supplementary Material online). For smaller SVs (<50 bp), accuracy was slightly lower, but still reached at least 91.5% for deletions and 92.9% for insertions across the divergent references. Correspondingly, both error and missing rates increased with sequence divergence but remained low overall: error rates were below 0.87% and missing rates below 1.26% for deletions; for insertions, error and missing rates were below 3.48% and 2.12%, respectively (Fig. 5e and 5f). Similar trends were observed in the tomato genome datasets (supplementary table S10 and fig. S8, Supplementary Material online). Collectively, these results demonstrate that SVGAP can accurately merge and genotype SVs across population-scale genome assemblies, even in the presence of moderate to high sequence divergence.

We also benchmarked SVGAP against several existing SV genotyping tools capable of handling population-scale genome assemblies—PGGB (Garrison et al. 2024), AnchorWave + TASSEL (Bradbury et al. 2007; Song et al. 2022), and Cactus (Hickey et al. 2024)—using the set of 20 simulated rice genomes in which SVs were introduced entirely at random based on the “Nipponbare” reference genome (see the “Methods” section). SVGAP demonstrated the best overall performance across all four evaluation metrics, achieving a recall rate of 99.91%, genotyping accuracy of 99.89%, a genotyping error rate of 0.11%, and a missing rate of 0.43% (supplementary fig. S9, Supplementary Material online). While PGGB showed slightly higher genotyping accuracy (99.99%) and a lower error rate (0.01%), it had a significantly lower recall rate (94.18%), indicating reduced sensitivity. AnchorWave + TASSEL also performed reasonably well, but none of its metrics surpassed those of SVGAP. Cactus showed the weakest performance across all metrics—even after testing two pipeline configurations—likely due to its current limitations when applied to large, structurally complex plant genomes.

### Discovering SVs in Maize Genomes and Uncovering Hidden Genomic Diversity in Its Centromeres

To assess the applicability of SVGAP in large and repetitive plant genomes, we applied it to 26 diverse maize genomes, including 25 nested association mapping (NAM) founder inbreds (Flint-Garcia et al. 2005) and the reference B73 (Hufford et al. 2021). The maize genome is known for its large size, approximately 2 Gb, with a significant proportion (∼85%) consisting of TEs (Ou et al. 2024 ). Our initial intraspecific comparison reveals that the average alignable portion in synteny between each query genome and the reference B73 accounts for approximately 56% of the entire genome (supplementary fig. S10a and b, Supplementary Material online). This suggests the existence of substantial PAVs within maize genomes, which makes them an excellent model for evaluating the effectiveness of SVGAP.

**Fig. 6.**
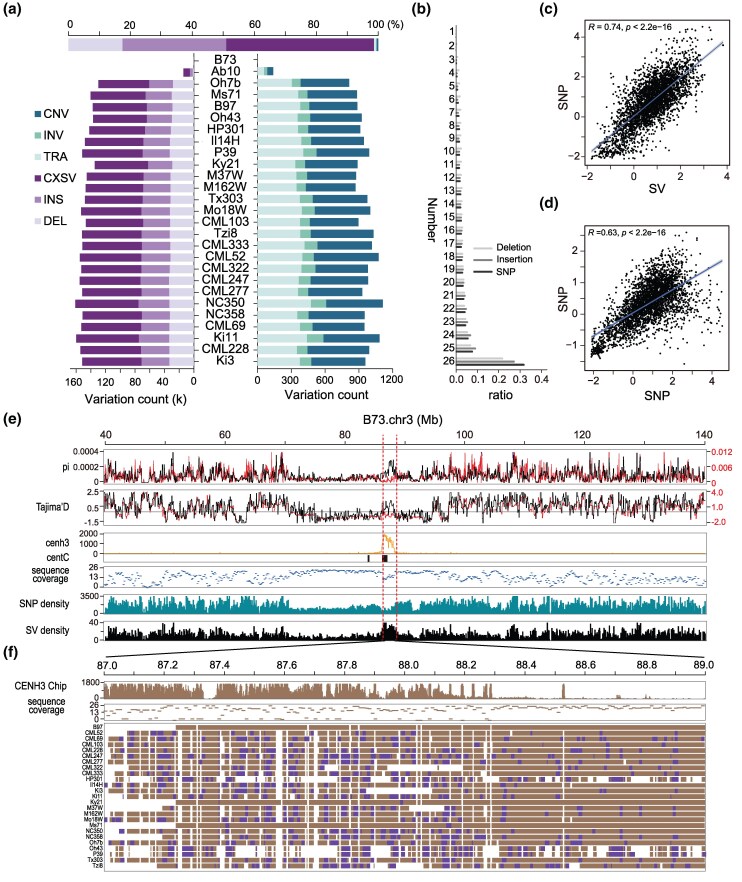
SV discovery in maize and hidden genomic diversity uncovered around its centromeric regions. a) SVs detected in 26 diverse maize genomes using SVGAP. b) Genotyping frequency for different types of genetic variations including insertion, deletion, and SNV across the samples. c) The plot graphs Tajima's *D* calculated based on SNP and SV, which detected by SVGAP across nonoverlapping 100-kb windows of the maize genome, with the line indicating the correlation, which is strongly positive (Pearson r = 0.74, *P* = 2.2 × 10^−16^). d) The plot graphs Tajima's *D* calculated based on SNP detected by SVGAP in 26 maize genomes and SNP from a prior study, which identified SNP from 1,515 accessions across nonoverlapping 100-kb windows of the maize genome. The line here too indicates a strong positive correlation (Pearson’s *r* = 0.63, *P* = 2.2 × 10^−16^); e) Features of genetic variations and their population properties around the centromeric regions of chromosome 3. Panels from up to down indicate the SVs (black line) and SNVs (red line) average pairwise diversity (π) for nonoverlapping 100-kb windows, the SVs (black line) and SNVs (red line) Tajima's *D* for nonoverlapping 100-kb windows, CenH3 ChiP-seq reads mapping; distribution of maize centromere-specific tandem repeat (CentC), sequence coverage for nonoverlapping 10-kb windows across the 26 maize genomes, SNV density, and SV density. f) The increased genomic diversity around the cen3 detected by SVs is attributable to the amplification of centromere-specific retrotransposons. Brown boxes represent alignment blocks, while blue boxes indicate intact CRM insertions.

The alignments obtained from minimap2, which were determined through benchmarking (supplementary table S11 and fig. S10c and d, Supplementary Material online), were used in combination with SVGAP to detect SVs. We compared each of the 25 NAM parents and the Ab10 line to the B73 reference, revealing a cumulative total of 1,758,685 SVs ≥50 bp in size. Count per genotype (25 NAM parents) ranged between 61,451 and 76,248, not counting complex loci (Fig. 6a and supplementary table S12, Supplementary Material online). Notably, SVGAP found a significant number of complex loci, where regions between conserved syntenic blocks did not align to each other, implying rapid sequence turnover in these regions. Merging SVs across all lines yielded a total of 513,336 uniquely located SVs (excluding complex loci), including 192,454 deletions, 312,187 insertions, 2,084 inversions, and 6,611 copy number variants (Fig. 6a and supplementary table S12, Supplementary Material online). These numbers of SVs are considerably higher than those reported in the previous study (Hufford et al. 2021) that primarily used long-read mapping methods to identify SVs. For deletions, approximately two-thirds of those reported in previous studies were also detected by SVGAP; however, nearly two-thirds of the deletions identified by SVGAP were novel and had not been reported previously (supplementary fig. S10e, Supplementary Material online). Of those additional deletion calls detected by SVGAP, we found that a significant portion (45%) were independently detected in at least two genotypes, suggesting a substantial number of false negatives based on the long-read mapping methods used previously. For insertions, SVGAP detected nearly nine times more events (251,807 vs. 28,009) than the previous study, likely reflecting the inherent bias of long-read mapping methods toward detecting deletions over insertions (Ahsan et al. 2023), while de novo assembly methods likely solve this issue. The missing insertions were randomly distributed along chromosomes (supplementary fig. S10f, Supplementary Material online). To assess whether the SVGAP calls were FPs, we also performed PCR validation on 40 SVs. PCR confirmed that 14 of 16 novel deletions (87.5%) and 17 of 24 novel insertions (70.8%) were accurate (supplementary fig. S11 and table S13, Supplementary Material online). Thus, SVGAP identifies bona fide maize SVs that had not been detected in previous work.

We next used SVGAP to genotype deletions and insertions across all samples and generated the full genotype VCF files for each. The SVGAP genotyping results revealed that 52.3% of deletions and 63.3% of insertions could be genotyped in at least 20 lines, compared to those observed for SNPs (63.2%) and multiple sequence coverage (Fig. 6b and supplementary fig. S10g and h, Supplementary Material online). We next evaluated the quality of the genotyping result by examining the population properties of SVs. Consistent with previous observations (Hufford et al. 2021), both SVs and SNPs were more prone to occur at the chromosomal ends than middle centromeric regions (supplementary fig. S12, Supplementary Material online). Furthermore, the genomic diversity calculated with SV and SNP data consistently revealed a reduction of diversity across most centromeres, consistent with previous work (Schneider et al. 2016). SV and SNP diversity were significantly correlated across chromosomal windows (Pearson’s *r* = 0.74, *P* < 2.2 × 10^−16^; Fig. 6c). Also, the SNP diversity calculated with SVGAP data from the 25 diverse maize genomes was significantly correlated across chromosomal windows compared to those calculated with high-quality SNPs from 1,515 maize accessions (Pearson’s *r* = 0.63, *P* < 2.2 × 10^−16^; Fig. 6d) (Grzybowski et al. 2023). Together, these results imply that SVGAP can effectively genotype SVs across large samples of plant genomes, including complex genomes like maize.

While there was a substantial correlation between SNP and SV diversity across the majority of chromosomal windows, certain regions, particularly within some centromeres, displayed noticeable disparities (Fig. 6e and supplementary fig. S12, Supplementary Material online). Centromeres are among the most dynamic parts of the plant genome (Wlodzimierz et al. 2023). Visually, centromeres and their flanking regions often display lower genomic diversity than chromosome arms in maize. Surprisingly, these regions contain highly conserved sequences in the population, which are reflected by the significantly higher sequence coverage (i.e. the number of lines that are alignable in synteny to B73 across the 25 maize lines) (Fig. 6e). We specifically focused on two centromeric regions (*Cen3* and *Cen9*) that showed high SV but not SNP diversity. The higher SV diversity observed in Cen3 (Fig. 6f) and Cen9 (supplementary fig. S13, Supplementary Material online) than their flanking regions is the result of a higher occurrence of SVs in these genomic regions. After a detailed examination of the SVs that occurred in these regions, we found most were insertions of centromere-specific retrotransposons in maize (CRMs). Of 459 SVs detected in the 2 Mb regions of centromere 3, up to 267 were CRM insertions (Fig. 6f). These findings demonstrate that frequent CRM insertions drive localized SV diversity and highlight SVGAP's ability to detect SVs in highly repetitive genomic regions, uncovering previously hidden genetic variation.

### Execution Time and Memory Efficiency

To evaluate the runtime and memory usage of each step in the SVGAP pipeline, we conducted tests using 49 rice genomes on a high-performance system. Specifically, we utilized a dual-CPU AMD EPYC 9654 96-core node running on Linux (Rocky Linux release 9.3), equipped with 1 TB of DDR5 RAM and connected to storage through 1 GB RAID controllers. After conducting the tests, we found that the SVGAP pipeline, which includes genome alignment, SV discovery, and genotyping steps, could be completed in around 2 days on our system. Additionally, the peak memory usage of our method remained below 150 GB. A summary of the computational resources is provided in supplementary table S14, Supplementary Material online, detailing the time and memory usage for each step of the SVGAP pipeline. These results indicate that SVGAP can efficiently leverage reasonable computational resources, making it suitable for routine SV discovery and genotyping in large assembly samples within a population or species.

## Discussion

Recent advancements in sequencing technologies and computational methods have made it possible to routinely generate nearly complete genome assemblies from large samples of the same or closely related species (De Coster et al. 2021). These assemblies provide valuable opportunities to investigate the full spectrum of genetic variants and their functional consequences, especially previously inaccessible ones, across diverse evolutionary contexts and genomic landscapes (Altemose et al. 2022; Li et al. 2023). Consequently, there is a growing demand for flexible and scalable computational tools to analyze population-scale genome assemblies, particularly in plant genomics, where the dynamic and plastic nature of plant genomes presents significant challenges for sequencing, assembly, alignment, and variant discovery (Murat et al. 2012; Reneker et al. 2012; Song et al. 2024).

In this study, we have introduced SVGAP, a versatile pipeline for population-level identification of SVs from large samples of genome assemblies. SVGAP identifies SVs through pairwise WGAs, comparing multiple genome assemblies against a reference genome. The pipeline includes tools for combining SVs, genotyping a unique call set across all samples to produce fully genotyped VCF files, and annotating SVs (Fig. 1). Benchmark analyses using simulated plant genomes of varying complexities—spanning individual, population, and phylogenetic levels—demonstrate that SVGAP consistently outperforms existing tools in SV discovery (Figs. 3 and 4). It also provides accurate population genotyping and exhibits robustness against false variant discoveries (Fig. 5). We believe that the accuracy and comprehensive SV data generated by SVGAP make it a useful resource for constructing reference pangenome graphs (Garrison et al. 2018; Hickey et al. 2020; Li et al. 2020a). While SVGAP is primarily designed for chromosome-scale assemblies within the same species, it can be applied to assemblies of varying quality, size, and divergence; however, lower assembly quality compromises SV detection performance (supplementary fig. S14, Supplementary Material online).

An optimal WGA is crucial for SVGAP and other similar SV callers. Selecting appropriate aligners for the species under examination is a prerequisite for ensuring the accuracy and comprehensiveness of SV discovery. Our initial assessment of existing aligners for large and repetitive plant genomes, such as maize and pepper, has uncovered significant challenges, because only a subset of aligners can effectively complete this task (Fig. 2). The practical obstacles encountered include long run times (e.g. Lastz and MUMmer4-default), high memory consumption (e.g. Minimap2 and Last-split), incomplete alignments (e.g. GSAlign), storage inefficiencies (e.g. Last-default), and difficulties resolving specific chromosomal arrangements (e.g. AnchorWave). One approach for aligning large plant genomes involves splitting chromosomes into smaller segments and aligning these segments individually to the reference genome. We demonstrated this strategy in maize and pepper using minimap2 for alignment within the SVGAP pipeline. Nevertheless, our results underscore the pressing need for new alignment tools optimized for plant genomes (Song et al. 2024).

Comparisons of SV callsets across different callers—or the same caller using different aligners—reveal substantial inconsistencies. Genome complexity plays an important role, because the level of inconsistency increases with the complexity of the genome (supplementary fig. S2, Supplementary Material online). Local sequence features are also critical, because inconsistent SV calls tend to overlap with repetitive regions and low-complexity sequences enriched in short tandem repeat (Jakubosky et al. 2020). There is also an underlying biological complication, in that regions flanking indels demonstrate elevated mutation rates and sequence diversity that varies according to mating system and divergence times (Tian et al. 2008; Hollister et al. 2010). Although these boundary-specific dynamics were likely not captured in our simulation approach, our results nonetheless accentuate that a consistent choice of callers and aligners is essential for comparative analyses. Additionally, the alignment of indel alleles presents a significant challenge in accurate SV detection. While some approaches may address this issue for simple and/or small indels (e.g. GATK's left alignment), the problem becomes more complex for larger and more complex SVs. Alignment software is inherently constrained by its underlying algorithms, which may not always reflect biological reality. This can lead to situations where biologically meaningful alignments cannot be reliably identified, potentially introducing biases in SV calling. Future advancements in alignment algorithms, incorporating more sophisticated models of genome evolution and structure, may help mitigate these biases and improve the accuracy of SV detection, especially in complex genomic regions.

Genome divergence is an important factor to consider in SV discovery. Our benchmarking analyses indicate that detection recall and accuracy progressively decline as divergence increases, while the false discovery rate rises. This is expected, because genome divergence often leads to synteny decay due to large-scale rearrangements and gain/loss mutations. This restricts variant detection to regions where sequences remain alignable between genomes. Consequently, ancient and newly arising variants, such as SVs, in unalignable regions become increasingly difficult to detect. Furthermore, reduced sequence identity—driven by the accumulation of SNVs and InDels—introduces ambiguity in breakpoint detection for SVs within alignable regions as divergence increases. Employing sequence divergence-aware aligners may further enhance the accurate and reproducible detection of SVs across divergent genomes (Li 2018; Song et al. 2022). Although the choice of reference genome can greatly influence the number of detected SVs—particularly when the reference is highly divergent— our results indicate that this effect may be limited when comparing accessions within the same species (supplementary fig. S15, Supplementary Material online).

While high-quality benchmark datasets with verified SVs exist for animals (Majidian et al. 2023), comparable datasets for plants are still lacking. Given the distinct genomic features of plants, creating such datasets is crucial for evaluating existing SV callers and developing optimized ones, though this process is often challenging and time-consuming (Sarkar et al. 2020). In this study, we developed an efficient simulation approach to partially address this gap by simulating SVs in genomes with varying divergence from a reference, preserving some of the characteristics of sequence divergence. Our evaluation has focused on SVs simulated in alignable regions during genome comparisons, but further studies with high-quality benchmark datasets or novel simulation applications will improve our assessment of existing methods and aid in developing new ones.

While our study focused primarily on insertions and deletions because of their prevalence, inversions and translocations are also crucial in structural variation analysis. SVGAP's alignment nets enable SV detection in rearranged genomic regions, as demonstrated by our identification of thousands of inversions and numerous complex loci. However, translocations are rare, and complex SVs are challenging to classify accurately, explaining their limited treatment in our current analysis. Future research should prioritize validating these inversions and potential translocations, given their potential impact on phenotypic variation and genome evolution.

Deciphering the mechanisms of SV formation is crucial after their discovery and can, in turn, guide the development of new tools. Understanding these mechanisms enhances SV detection accuracy, improves functional annotation, and offers insights into their evolutionary and biological significance. SVGAP has addressed simpler mechanisms like tandem duplications and TE or gene-related insertions, but further improvements are needed to resolve mechanisms that lead to more complex SV events, e.g., SVs that encompass multiple breakpoints, as well as nested TE insertions, which are prevalent in plant genomes (Sigman and Slotkin 2016). Nonetheless, SVGAP identified centromere-specific LTR-retrotransposon insertions across the maize NAM lines, illustrating regions where SV diversity is unexpectedly high relative to SNP diversity (Fig. 6e).

While the primary aim of SVGAP is to generate complete and accurate reference-based variant calls from large samples of high-quality genome assemblies, its output can also be valuable for pangenome construction. The resulting VCF files can be directly used to create VCF-derived pangenome graphs, such as those implemented in vg (Hickey et al. 2020) and Paragraph (Chen et al. 2019), and for further genotyping with these tools. The strategy for WGAs of large and repetitive plant genomes has potential implications for pangenome construction using tools based on WGAs or multiple genome alignments, such as PGGB (Garrison et al. 2024), PSVCP (Wang et al. 2023), and Minigraph-Cactus (Armstrong et al. 2020; Li et al. 2020a; Hickey et al. 2024). Given the computational challenges posed by large, repetitive, and complex plant genomes, additional efforts may be needed to optimize these tools for plants.

## Methods

### Overview of SVGAP

SVGAP is designed to identify SVs (≥50 bp), InDels (<50 bp), and SNVs from whole-genome pairwise assembly–assembly alignments between a reference genome and multiple query genomes. These alignments can be generated using tools such as minimap2 (Li 2018), MUMmer (Marçais et al. 2018), Last (Kiełbasa et al. 2011; Frith and Kawaguchi 2015), AnchorWave (Song et al. 2022), and others. SVGAP takes alignments from these external tools as input and follows a multistep workflow consisting of six main steps (Fig. 1): (i) alignment files are converted into the desired format for SVGAP, (ii) Construction of syntenic and orthologous alignments, (iii) SV discovering between each query genome and the reference, (iv) merging SVs across all pairwise comparisons, (v) SV regenotyping by extracting local alignments from WGAs, and (vi) SV annotation. The output of SVGAP consists of standard VCF files that contain confidently called SVs and other variants.

#### Conversion of Alignment Files

Alignment files from external aligners must be converted to AXT format (https://genome.ucsc.edu/) for downstream processing with SVGAP. MUMmer's output *.delta* files are converted using the delta2maf (Delcher et al. 2002) and mafToAxt (https://hgdownload.soe.ucsc.edu/admin/exe/linux.x86_64/) programs. Minimap2's output *.paf* files are converted using paftools.js (distributed with Minimap2) and mafToAxt, with minor modifications required for the intermediate *.maf* files. Output *.maf* files from AnchorWave, Last, and Lastz are also converted using mafToAxt, with additional adjustments needed for compatibility. Other tools involved in the conversion process include maf_sort and maf_order from TBA (Blanchette et al. 2004), along with scripts developed for SVGAP in this study.

#### Construction of Whole-Genome Synteny Alignment

The converted alignment files were processed into structures known as chains and nets (https://genome.ucsc.edu/) with a series of programs developed by Kent et al. (2003). Genome FASTA files were converted to 2bit format with faToTwoBit, and chromosome length files were generated using faSize with the -detailed parameter. Chains were constructed using axtChain with the -linearGap = medium option and filtered with chainPreNet. Nets were created using chainNet with the -minSpace = 1 option and subsequently annotated with netSyntenic. The resulting *.synnet* files were converted to pairwise*.maf* files using netToAxt and axtToMaf, which were then used to generate *.maf* files with single coverage in both target and query genomes using single_cov2 from the TBA program (Blanchette et al. 2004).

#### Variant Detection

SVs and InDels are identified for each query genome relative to the reference genome using the *.synnet* files with the program *PairASSYSV.pl*. These files can be further filtered with *SynNetFilter.pl* to enhance accuracy, although this may result in a slight loss of sensitivity. This step is particularly recommended when the compared genomes exhibit significant sequence divergence and rearrangements, ensuring that variants are identified only from reliable alignment portions, specifically syntenic and orthologous genomic regions, typically represented as top chains (the highest-scoring chained alignments). Gaps within the top chains may correspond to insertions, deletions, inversions, duplications, or translocations. Therefore, variants may also be retrieved from lower-scoring chains (i.e. secondary chains) if they fill large gaps in the top chains and indicate inversions or translocations (supplementary fig. S1, Supplementary Material online). SVGAP currently reports six types of SVs: insertions, deletions, tandem duplications, inversions, translocations, and complex events. Additionally, SNVs are jointly called from pairwise single-coverage *.maf* files using *SNPgenotyping.pl*. All aforementioned Perl scripts are included in SVGAP.

#### SV Merging

SVGAP independently identifies SVs for each sample relative to the reference genome. Due to sequence divergence, the same SV event from different samples may exhibit slight differences in their breakpoint coordinates. To create a nonredundant SV dataset, SVGAP uses the program *Combined.pl* to merge SVs across samples. SVs from all samples are first combined for each type and sorted based on reference coordinates. For deletions, inversions, and translocations, events with identical coordinates or reciprocal overlap of at least the specified cutoff threshold (default 90%) are merged into a single event. For insertions and duplications, events with a breakpoint shift range of no more than 12 bp (or as defined by the user), inserted lengths varying by no more than 20% (adjustable), and sequence identity above 50% (adjustable) are considered a single event.

#### SV Regenotyping

The merged SV dataset lacks complete genotyping information, as only samples where the event was reported have clear genotypes, while the status in samples that did not report the event remains uncertain. This uncertainty may arise from sequence gaps, sequence loss due to divergence, or genotypes identical to the reference. To address this issue and obtain a comprehensive genotype status across samples, SVGAP provides DELgenotyping.pl, INSgenotyping.pl, and InDelGenotyping.pl to conduct a second round of genotyping for deletions, insertions, and InDels, respectively (see supplementary fig. S6, Supplementary Material online). These tools extract and analyze the local alignment around each SV locus from pairwise WGAs to infer the genotype status (e.g. reference type, alternative type, or missing data) and generate a standard VCF file for each SV type. For deletions and InDels, local alignments extracted from the single-coverage .maf file are directly examined to verify the target event. For insertions, local sequences around the SV locus are first extracted from each query genome and realigned to the reference sequence using Stretcher (Myers and Miller 1988). The resulting alignment is then used to infer the insertion event based on SV length and sequence identity.

#### SV Annotation

To annotate SVs, SVGAP requires user-provided FASTA files containing full-length coding DNA sequences (CDS) and TE sequences for the species of interest. Rather than comparing SV coordinates directly with annotation files (e.g. GFF), SVGAP extracts the sequences of deletions and insertions from VCF files and aligns them to the CDS and TE libraries using minimap2. This approach enables the identification of gene-related deletions/duplications and TE insertions based on an adjustable sequence overlap rate (e.g. 50%). Additionally, SVGAP identifies local tandem duplications by comparing inserted sequences to their immediate 5′ and 3′ flanking regions; insertions sharing ≥50% size overlap with either flank are classified as tandem repeats. All annotation steps are carried out by the *SVannotation.pl* script. The TE library can be generated using ethylenediaminetetraacetic acid (Ou et al. 2019).

### Assessing and Selecting Aligners for Plant WGA

We selected 14 sequence aligners to evaluate their effectiveness in aligning plant genomes. These tools can be categorized into three groups: (i) aligners designed for genome-scale alignments, including Lastz (Harris 2007), Last (Kiełbasa et al. 2011; Frith and Kawaguchi 2015), MUMmer4 (Marçais et al. 2018), GSAlign (Lin and Hsu 2020), and AnchorWave (Song et al. 2022); (ii) aligners for long-read mapping and alignment, such as minimap2 (Li 2018; Song et al. 2022), MECAT2 (Xiao et al. 2017), Blasr (Chaisson and Tesler 2012), BWA-MEM (https://arxiv.org/abs/1303.3997), Ngmlr (Sedlazeck et al. 2018), and GrapMap (Sović et al. 2016); and (iii) aligners optimized from minimap2 for specific targets, including Pbmm2 (https://github.com/PacificBiosciences/pbmm2/), Unimap (https://github.com/lh3/unimap), and winnowmap2 (Jain et al. 2022). To evaluate these aligners, we aligned two rice genomes, MH63 and ZS97 (Song et al. 2021), using their default or recommended parameters. Their performance of these aligners is detailed in supplementary note S2, Supplementary Material online.

Based on the evaluated results, we selected six aligners for further evaluation: Last, Lastz, minimap2, MUMmer4, GSAlign, and AnchorWave. These aligners were used to align two individual genomes from five additional species: human (CHM13 [Nurk et al. 2022] vs. YAO [He et al. 2023]), fruit fly (iso-1 [Hoskins et al. 2015] vs. A4 [Chakraborty et al. 2018. 2019]), maize (B73 [Hufford et al. 2021] vs.Mo17 [Chen et al. 2023c]), tomato (SL5.0 vs. TS60 [Zhou et al. 2022]), and pepper (CaT2T [Chen et al. 2024] vs. Zunla-1_v3.0 [Zhang et al. 2025]). In total, we tested 11 aligner–parameter combinations: minimap2 with four settings (-asm5, -asm10, -asm20, and default), MUMmer4 with two options (-maxmatch and default), AnchorWave with -genoAli and -proAli modes, and Last, Lastz, and GSAlign using their default configurations. Minimap2, MUMmer4, AnchorWave, and GSAlign were executed with 24 threads, whereas Last and Lastz used default configurations without specified thread counts.

We evaluated the aligners across species based on runtime, peak memory usage, storage requirements for raw alignments, and the proportion of the reference genome covered by alignable regions. Runtime and peak memory usage were measured using the command “/usr/bin/time –v.” Storage requirements were calculated from the size of the *.maf* file, which was converted from the default output format generated by each aligner. The proportion of the reference genome covered by alignable regions was calculated from single-coverage *.maf* files using a custom Perl script. The results are summarized in supplementary table S3, Supplementary Material online.

Specifically, SVGAP includes the program *SplitFa.pl*, which divides large chromosomes into smaller segments with user-defined sizes and step intervals. We applied this split-genome approach to large and repetitive plant genomes (e.g. maize and pepper) when testing minimap2, as it often encounters memory exhaustion issues. This strategy effectively reduces peak memory usage. The coordinates of the segments in their original genomic positions can later be restored using the *Convert2Axt.pl* program.

### SV Simulation and Generation of Benchmark Datasets

We generated three benchmark datasets with simulated SVs for rice, tomato, and fruit fly to provide ground truth for evaluating SV detection across different aligners and callers. The first dataset was created by introducing SVs into the respective reference genomes using RSVSim (Bartenhagen and Dugas 2013). For rice (Nipponbare; Kawahara et al. 2013; Shang et al. 2023), we simulated 20,328 deletions and 10,046 insertions (≥50 bp). For tomato (SL4.0; Hosmani et al. 2019), we simulated 15,245 insertions and 20,328 deletions (≥50 bp). We also simulated 7,264 deletions and 4,290 insertions on the fruit fly iso-1 genome. We refer to these as reference-based simulated datasets.

The second dataset was generated by simulating SVs among genomes at varying levels of divergence, based on the phylogenies of *Oryza* and *Solanum*. We refer to these as phylogeny-based simulated datasets. For *Oryza*, we independently simulated 10,000 deletions (ranging from 50 bp to 20 kb) and 10,000 insertions (ranging from 50 bp to 40 kb) using RSVSim across eight phylogenetically divergent genomes, representing approximately 6.76 million years of divergence (Stein et al. 2018). These genomes include five from *O. sativa*: MH63 (Song et al. 2021), R498 (Qin et al. 2021), 9311 (Qin et al. 2021), ZS97 (Song et al. 2021), and Nipponbare (Shang et al. 2023), along with three from other species: *O. rufipogon* (IRGC106523) (Zhou et al. 2023), *O. glaberrima* (CJ14) (Qin et al. 2021), and *O. punctata* (IRGC105690) (Zhou et al. 2023). For *Solanum*, we also selected eight phylogenetically divergent genomes and independently simulated 15,000 deletions and 15,000 insertions (ranging from 1 bp to 50 kb) in each one. These genomes include three from *Solanum lycopersicum*: SL4.0 (Hosmani et al. 2019), M82 (Alonge et al. 2020), and ZY65 (Li et al. 2023), as well as five from closely related species: *Solanum galapagense* (ZY56), *Solanum pimpinellifolium* (ZY57), *Solanum chmielewskii* (ZY60), *Solanum peruvianum* (ZY61), and *Solanum habrochaites* (ZY59) (Li et al. 2023). These tomato genomes span approximately or less than 7.5 million years of divergence (Li et al. 2023), with the proportion of sequence alignable to the reference SL4.0 ranging from 41.5% to 100% (supplementary fig. S4a, Supplementary Material online). This proportion was calculated from each single-coverage *.maf* file between the query genome and the SL4.0.

The third dataset was generated by simulating SVs in population-scale genome assemblies for rice and tomato. A program called *Simulator_pop.pl* was developed to introduce three types of SVs—deletions, insertions, and duplications—into the reference genomes, allowing for the simultaneous generation of multiple genomes. This program requires three inputs: a reference genome, a set of SVs, and the number of genomes you wish to generate. The set of SVs should include the following information: coordinates, genotypes across samples (where “0” indicates similarity to the reference and “1” denotes an alternative), and sequences for the insertion events. We prepared the set of SVs for both rice and tomato using real datasets from previous studies (Qin et al. 2021; Liao et al. 2022), with coordinates reassigned. For simplicity, these SVs were randomly selected from four chromosomes (Chr01, Chr03, Chr08, and Chr12 in rice; Chr01, Chr02, Chr03, and Chr04 in tomato), along with a subset of TE-associated insertions. In total, there are 2,977 deletions, 2,232 insertions, and 139 duplications for rice, and 3,017 deletions, 3,382 insertions, and 152 duplications for tomato, with sizes ranging from 50 to 10,000 bp. We simulated 21 genomes for each species based on the reference genome Nipponbare for rice and SL4.0 for tomato. In each simulated genome, approximately half of the SVs are present, while one genome contains all the SV events. We refer to these as population-based simulated datasets.

### SV Overlapping Analysis

To assess the consistency of SV calls obtained from different aligners and callers, we compared the overlap of SV calls across various methods. We applied the following approach to generate a data frame for the UpSet plot (see Fig. 3a): (i) SVs (deletions and insertions) detected under different methods were combined and categorized into three size groups: less than 5 Kb, 5 to 10 Kb, and larger than 10 Kb, for each type; (ii) SVs in each size group were merged independently using BEDTools (v2.30.0) to remove redundancy; (iii) the three size groups were then combined to create a unified reference SV set; (iv) SVs identified by each method were compared against the reference set to determine their overlap. An SV was considered a positive call if its coordinates had 100% overlap with any SV in the reference set. This approach provides a comprehensive list of unique SVs generated from all methods and their detectable status across those methods. The UpSet plot was generated using TBtools-II (Chen et al. 2023a).

We also created a similarity matrix based on the pairwise overlap rate among methods. The pairwise overlap between methods was calculated as the proportion of SVs exhibiting at least 80% reciprocal overlap. We used the Jaccard index distance to generate cluster groups and assess the agreement (see Fig. 4c) between methods using TBtools-II (Chen et al. 2023a).

### Benchmarking Aligners for SV Detection With SVGAP

Pairwise WGAs generated using AnchorWave (v1.0.1), Minimap2 (2.24-r1122), MUMmer (version 4), GSAlign (v1.0.22), Last (version 1406), Lastz (version 1.04.22), Unimap (0.1-r41), and Winnowmap (version 2.03), with default or recommended settings (supplementary table S2, Supplementary Material online), were used as inputs for SVGAP to detect SVs. We benchmarked the aligners using the first two simulated datasets (see the “SV Simulation and Generation of Benchmark Datasets” section) for rice and tomato, as well as a reference-based simulated SV dataset for *Drosophila*. For tomato and *Drosophila*, we excluded Unimap and Winnowmap from the comparison because their performance did not outperform Minimap2 in our analysis of rice.

The SV call sets obtained from SVGAP using different aligners were compared against the ground-truth SV set to assess recall, precision, and F1 score. An SV call (deletion or insertion) was considered a true positive (TP) if it exhibited at least 80% reciprocal overlap with the corresponding call in the ground-truth SV set. For the reference-based simulated dataset, SVs were identified between the simulated genome (denoted as A') and its original reference genome (denoted as A). In contrast, for the phylogeny-based simulated dataset, two SV datasets were generated for each comparison. The first dataset was identified between the reference genome (A) and the original query genome (B), serving as the background. The second dataset was identified between the reference genome (A) and the simulated query genome (B'). The comparison SV dataset was derived by subtracting the first dataset from the second, which was then used to evaluate against the ground-truth SV set for calculating recall, precision, and F1 score. Detailed formulas for these calculations are shown in Fig. 3d, and the specific commands and scripts are provided in supplementary note S2, Supplementary Material online.

### Benchmarking Callers for SV Detection

We benchmarked SVGAP alongside seven popular assembly-vs.-assembly SV callers: AnchorWave (v1.0.1), SyRI (v1.6), SVIM-asm (version 1.0.3), Assemblytics (https://github.com/MariaNattestad/Assemblytics), GSAlign (v1.0.22), MUM&Co (v3.8), and paftools (used with minimap2, 2.24-r1122) for SV detection, utilizing the same benchmark simulated SV datasets previously employed for benchmarking callers in both rice and tomato. AnchorWave was executed with the “-v” option to generate SV calls. For SyRI, alignments from MUMmer4 and minimap2 were employed to call SVs. SVIM-asm utilized alignments from minimap2 with the options “-a -x asm5 –cs -r2k” for SV calling. GSAlign was used for SV calling based on its alignment process. Assemblytics relied on alignments from MUMmer4.0 with the options “–maxmatch” and default settings. For MUM&Co, alignments from MUMmer4.0 with default options were used for SV calls. Paftools was invoked alongside minimap2. All methods were executed with default or recommended parameters, as detailed in supplementary table S7, Supplementary Material online. The resulting SV callsets were compared against the ground-truth SV set to calculate recall, precision, and F1 score using the methods described in the previous section.

### Benchmarking SV Genotyping for Population-Scale Genome Assemblies

To evaluate SVGAP's performance in SV genotyping across population-scale datasets, we conducted benchmarking analyses using simulated genome assemblies of rice and tomato. SVGAP performs genotyping in two key steps: (1) merging SVs from all individuals to generate a nonredundant, population-wide SV callset, and (2) regenotyping each SV in individual genomes by revisiting WGAs against the reference. We assessed each of these steps separately. For this purpose, we simulated genome assemblies for 21 individuals in both rice and tomato by introducing three types of SVs—deletions, insertions, and duplications—into their respective reference genomes (Nipponbare for rice and SL4.0 for tomato), as described in the previous section.

The simulated genome assemblies constructed at the population scale were then individually aligned onto the reference genome, along with seven other divergent genomes, exhibiting varying levels of phylogenetic distance, as previously described. This was accomplished using MUMmer4 with its default parameters. These alignments were input into SVGAP for detecting SVs between each simulated individual and their corresponding reference genomes. SVGAP then combines SVs from all individuals to produce a unique set of SVs. The SV callset contains not only the simulated SVs but also a set of preexisting SVs between the reference and other genomes, which should be filtered out. Any SVs in the callset with a genotype frequency greater than 19/21 were considered real SVs and were excluded, except when the assemblies were mapped to the reference where they originated. Post filtration, any called SVs with a simulated target match (i.e. minimum reciprocal overlap of 50%) were classified as TPs, while those without a match were deemed FPs. Evaluation metrics used to measure the first combination step included completeness, calculated as the percentage of TP divided by the total number of simulated SVs times 100%; accuracy, calculated as the percentage of TP divided by the total number of all predicted SVs; and error rate, calculated as the percentage of FP divided by the total number of all predicted SVs.

For the TP SV calls, we performed an assessment of genotyping accuracy, error rate, and missing rate for each individual. Accuracy was quantified as the percentage of correctly genotyped SVs out of the total reported SVs. Similarly, the error rate represented the percentage of incorrectly genotyped SVs among the total reported SVs. Additionally, the missing rate was determined as the percentage of ungenotyped SVs compared to the total reported SVs. More details are described in supplementary methods, Supplementary Material online.

### Comparison to Other Genotyping Pipelines

We compared SVGAP with three other SV discovery and genotyping tools suitable for population-scale genome assemblies: PGGB, Cactus, and AnchorWave combined with TASSEL. For this comparison, we simulated 4,517 deletions (≥50 bp) and 3,258 insertions (≥50bp) on four chromosomes of the Nipponbare rice genome to generate a panel of 20 rice genomes, each carrying approximately half of the simulated SVs. Details on how these tools were applied are provided in the supplementary methods, Supplementary Material online. All pipelines were benchmarked on the same simulated dataset, and their performance was assessed using the metrics shown in Fig. 5c.

### SV Discovery Using SVGAP in Maize Genomes and Subsequent Analysis

Benchmarks for SV identification with different aligners were first conducted with simulated SVs. We used B73 (version 5) as the reference genome, while the other 25 NAM lines and the A10 assembly served as query genomes for SV identification. Each query genome was initially split into 20-Mb segments with a 2-Mb overlap using the *SplitFa.pl* program. These segments were then aligned to the B73 genome using minimap2 (2.24-r1122) with default settings to minimize memory usage. The coordinates of the segments were converted back to their original genomic coordinates using *Convert2Axt.pl*. Following the SVGAP workflow, we called SVs and other genomic variants. The output includes VCF files for deletions and insertions (greater than 50 bp) as well as SNVs.

The VCF files were used to calculate the genotyping rate across samples for each variant, indicating how many samples could be genotyped for that variant. For each variant, the number of samples with a valid genotype (i.e. 1/1 or 0/0 in the corresponding VCF file) was counted. Tajima's *D* and nucleotide diversity (π) were calculated in nonoverlapping 100-kb windows along the chromosomes based on VCF files for SVs and SNVs using VCFtools (v0.1.16). An additional SNP VCF file from 1, 515 maize accessions was used for comparison, which was obtained from a previous study (Grzybowski et al. 2023) and downloaded from https://www.maizegdb.org/. SNP and SV densities were also calculated for nonoverlapping 100-kb windows along the chromosomes. Sequence-alignable coverage was defined as the number of query genomes aligned to the B73 reference genome over at least half the length of each fixed window. This metric was calculated for each 10-kb window from the single-coverage .maf file for each sample.

Maize CENH3 ChIP-seq data from a previous study (Schneider et al. 2016) was downloaded from GenBank (accession no. SRP067358). The Illumina paired-end reads were mapped to the B73 reference genome (version 5) using Bowtie with the following parameters: -X 2000 –chunkmbs 3000 -k 3 –strata –best -v 2 -q.

### Runtime and Memory Requirements

We evaluated each step of the SVGAP pipeline using 49 rice genomes (Zhou et al. 2020; Qin et al. 2021; Song et al. 2021) on a high-performance computing system. The system consisted of a dual-CPU AMD EPYC 9654 96-core node running Rocky Linux 9.3, equipped with 1 TB of DDR5 RAM and connected to storage via 1 GB RAID controllers. Runtime and memory usage were monitored using the Linux time -v command. Genome alignment was performed with MUMmer4.0 using default settings.

## Supplementary Material

msaf180_Supplementary_Data

## Data Availability

All data used in this study are publicly available and cited in the appropriate places where mentioned. SVGAP and user manuals are publicly available at http://github.com/yiliao1022/SVGAP under the MIT License. We used the v1.0 version for SV discovery and benchmark in the manuscript. Key custom Perl scripts used in the manuscript can be found at http://github.com/yiliao1022/SVGAP/Utils.
